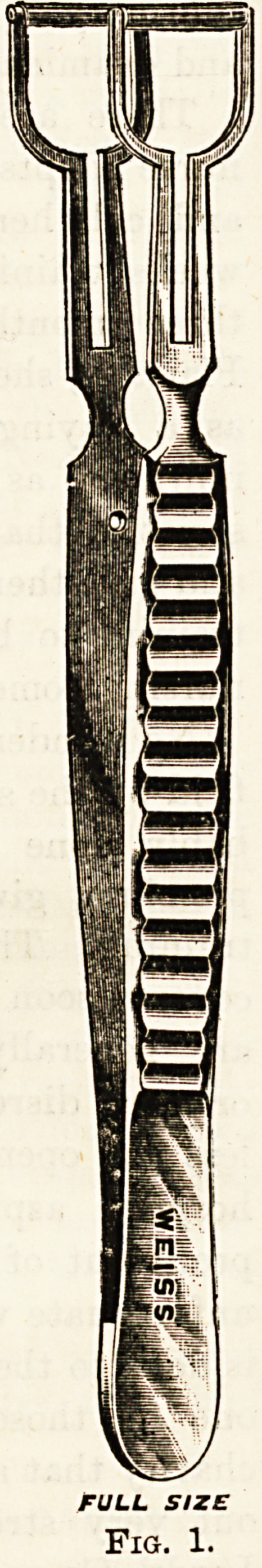# The Hospital. Nursing Section

**Published:** 1903-07-11

**Authors:** 


### The Hospital.

### Nursing Section.

Contributions for this Section of "The Hospital" should be addressed to the Editor, "The Hospital" Nursing Section, 28 & 29 Southampton Street, Strand, London, W.C.

No. 876.—Vol. XXXIV. *SATURDAY, JULY* 11, 1903.

### Notes on News from the Nursing World.

### NURSING AT THE FRENCH HOSPITAL.

The system of nursing at the French Hospital, which was visited by the President of the French Republic on Tuesday, is, of course, on French lines. The work is performed entirely by the Sisters of the Sacred Heart, whose convent is at Versailles. There are no ward maids, and the Sisters do everything except the polishing of the parquet floors. This is the duty of a man who is known as "the infirmier." No salaries are paid, the services of the Sisters being freely given to the hospital, but an allowance of £12 a year is made to each of them. They are 15 in number, including the Sister Superior. There is a Sister in charge of the theatre and the maternity department, another looks after the linen room, two superintend the cooking, and upon the remaining 10 devolves the whole of the medical and surgical nursing. Two sisters are always on night duty in charge of the 70 beds.

### LEVELLING UP IN WORKHOUSE NURSING.

Dr. Milson Rhodes is angry with the Workhouse Infirmary Nursing Association for daring to suggest that a nursing sub-department of the Local Government Board should be formed. But this is not a reason for misrepresenting the proposals which were made at the conference last week. It was not, as Dr. Rhodes asserts, urged that the Local Government Board should thrust nurses of their own selection upon such Boards of Guardians as Kensington, Liverpool, Birmingham, or Manchester. The sub-department which this Association has in view, would, as we gather, intervene only in the case of Poor-law Guardians who cannot, or will not, supply properly trained nurses for the infirmaries. We certainly do not wish to interfere with the great training schools in several London infirmaries and the provinces, but to level up the whole system of workhouse nursing to their standard. It is solely because the creation of the "qualified nurse" proposed by the Departmental Committee would have the reverse effect that we oppose it, and rejoice that the Workhouse Nursing Association makes it the central object of attack. Whatever means may or may not be employed to improve the present position, it is impossible to resist the conclusion of the Association that the best way to ensure sufficient candidates for nursing posts under the Local Government Board is to place Poor-law nursing generally upon a higher basis and to clearly define its responsibilities.

### ORDERLIES AND JEWISH INCURABLES.

The Princess Louise, on the occasion of the opening by her Royal Highness of the new building of the Home and Hospital for Jewish Incurables at South Tottenham last week, took tea with the matron, Miss Frances Cohen. Part of the nursing at this hospital is done by orderlies who have received their training in military hospitals, either at home or abroad. They attend on the 25 male patients in the home, three being on duty in the day and one at night. All the orderlies but the one who is in charge during the day and responsible for the whole of the nursing of the males, take their share of night work. The female patients, 30 in number, are nursed by one experienced charge nurse, who has received her training in a general hospital, with nine probationers under her. Two of these are on night duty by turn for two months. The feature of the work is the lifting, so many of the patients being paralysed, and they are dressed and carried into the sitting-room every day.

### ASSOCIATION FOR PROMOTING THE TRAINING AND SUPPLY OF MIDWIVES.

The Archbishop of Canterbury has accepted the office of President of the Association for Promoting the Training and Supply of Midwives, and Lady Balfour of Burleigh, Lady Mary Glyn, Lady Laura Ridding, Lady McLaren, Mrs. Randall Davidson, Mrs. Schwann, Sir J. Batty Tuke, M.D., M.P., and Dr. Cullingworth have consented to become vice-presidents. This organisation is an old friend under a new name, the Association for Promoting the Registration of Midwives, which did such excellent service in helping to secure the passing of the Midwives Act, having been reorganised. It now appeals to all former supporters and subscribers for assistance towards the practical outcome of the work in the past, namely, the providing of a small training home in a large, poorly-populated district, where help is absolutely necessary to provide skilled attendance for poor mothers. The cost of the home is estimated at £100 a year, and besides this there will, of course, be the necessary secretarial and postal expenses, if the association is to become a centre of information on all points connected with the training and supply of midwives, and a means of furthering similar schemes throughout the kingdom. It will work in co-operation with existing agencies which are concerned with the same object, and it is hoped that in addition to most of the members of the former executive committee, who have consented to serve again, representatives of leading nursing associations will join the council. All inquiries should be addressed to the secretary, Miss Maule, Attair, Ealing, W., and subscriptions should be sent to the hon. treasurer, Miss Grant, 22 Kensington Mansions, S.W.

### LADY HERMIONE BLACKWOOD AS DISTRICT NURSE.

IN the sixth annual report of the Bangor and District Nursing Society reference is made by the committee to the valuable help given by Lady Hermione Blackwood. When the district nurse was away on her holiday last summer Lady Hermione undertook district work in Bangor, and wherever she went she won the gratitude and affection of her patients. She has now a district of her own in Ireland, including Clandeboye, Helen's Bay, and Crawfordsburn. The President of the Bangor Society, the Dowager Marchioness of Dufferin and Ava, still continues to take the most active and kindly interest in the movement.

### THE MATRON OF BETHNAL GREEN INFIRMARY.

WE are glad to learn that the Guardians of Bethnal Green have declined to accept the resignation of the matron of the infirmary. Unfortunately, Miss Hopper is out of health, and on that account only she intimated to the infirmary committee her wish to resign. But the guardians, mindful of the admirable manner in which she has organised the nursing staff, and of the general excellence of her work, instead of accepting her resignation, have granted her two months' leave of absence in addition to her annual leave of a month. It is hoped that at the expiration of the three months she may feel strong enough to resume her duties.

### "QUEEN'S" NURSES AT RICHMOND.

ON the 20th of this month a public meeting will be held at Richmond, Surrey, for the purpose of supporting the enlargement of the St. John Ambulance Nursing Guild and its affiliation to the Queen Victoria's Jubilee Institute for Nurses. At a preparatory meeting a few days ago, some interesting details of the progress and position of the district nursing movement in Richmond were given. As the result of several generous gifts, notably that of £400 by Miss Catherine Onslow, a home has been provided for the Guild, and it is now possible to employ additional nurses. Miss Onslow very wisely urged that only Queen's nurses should be engaged in future, and it was decided that this course should be pursued, so that the Richmond District Nursing Association and Guild—the new name of the organisation—becomes a branch of the Jubilee Institute. At the forthcoming meeting the importance of increasing the income sufficiently to enable the committee to carry out the approved scheme, will be emphasised, and the project has already been taken up with vigour and enthusiasm by the Rev. Max Binney, vicar of Richmond, Dr. Boulter, Dr. Macguire, Dr. Bateman, and numerous ladies.

### TOTAL ABSTAINERS AT SURREY HOUSE.

A LARGE gathering of nurses assembled on Thursday afternoon at Surrey House, by the invitation of Lady Battersea, who gave to all present a most gracious and friendly welcome. Lady Battersea explained that the Nurses' National Total Abstinence League, under whose auspices the meeting had been arranged, was formed in order to gather members of the nursing profession together to fight intemperance wherever their work might lie and expressed the hope that new members might be gained that afternoon. Sir Thomas Barlow, Bart., next gave a practical address on the subject of the effects of alcohol on the human system and advised total abstinence from the use of it in the strongest terms. The Hon. Mrs. Eliot, president of the League, thanked Sir Thomas Barlow on behalf of the audience not only for the instructive address which he had given, but also for his kindness in sparing to the nurses so much of his valuable time. Mrs. Terrell expressed on behalf of all present their most grateful thanks for the very kind way in which both Lord and Lady Battersea had received them. During the afternoon a programme of songs, recitations, and 'cello solos was gone through, and after tea and strawberries, many of the beautiful contents of Surrey House were described by Lord Battersea, who took guests round the magnificent rooms.

### THE STIVEN MEMORIAL FUND.

THE final list of subscriptions to the Stiven Memorial Fund has now been issued, and we observe that it reaches a total of £1,002. This sum, which will serve as a lasting memorial to the late Dr. Stiven, will be immediately handed over, less expenses, to trustees, to be administered by them towards the maintenance of nurses for the poor in their own homes in the district of Harrow, a rapidly increasing proportion of the inhabitants.

### A SUCCESSFUL GARDEN FÊTE.

THE financial outcome of the garden fête in aid of the Strood and Frindsbury Nursing Association is one on which everyone connected with it is to be warmly congratulated. The fête took place in the grounds of Ravenswood, Frindsbury, was opened by Lady Cranborne, and lasted for two days, yielding the handsome sum of £240. Lest it should be supposed that to achieve this happy result, only a little work was needed, it may be stated that the fête was not recently thought of, but was first mooted at Christmas, when it was found that there was a serious deficiency in the income of the Nursing Association. All classes in the town were then invited to co-operate in the movement, and all worked with a will. Happily too, the weather was favourable, and the labour spent on the transformation scene in the beautiful grounds of Ravenswood, the residence of the secretary, was not in vain.

### DISTRICT NURSES IN JERUSALEM.

THE Bishop in Jerusalem makes the interesting statement that he is just starting a home for district nurses. Scientific nursing, Dr. Blyth says, as it is understood in England, is quite unknown amongst the natives in Jerusalem, and he mentions that an English doctor recently informed him that out of every five children who are born in the city no less than four die in their infancy. The Bishop is confident that many native women in his diocese will welcome the aid of English nurses, and that by their agency much may be done to bring wholesome influence and example into the homes of the Arabs. We are glad to learn that the funds for this work are already provided.

### HOUSEKEEPING AND NURSING.

IN the United States, as in England, the importance of nurses possessing a practical knowledge of kousekeeping is being realised. Thus, an American nursing organ affirms that people do not always send for a nurse because she can do better for their sick, but because the whole family is worn out with waiting and watching, and that then it becomes necessary for her to take the whole burden of work and nursing on her own shoulders and give the tired family a complete rest. The mistress of an ordinary household in the country town, it is said, hails with joy the nurse who has not become too "professional" to make herself really useful in case of sickness in families; and it is contended that there is a broad field for nurses who will cook, keep house, care for the children, and nurse the mistress when she is ill. As to any feeling that such work is derogatory, it is observed that the mistress of the household would do such things herself, and does not see why another woman, because she calls herself a nurse, should not be willing to do it also.

### PROGRESS AT PECKHAM.

The Peckham Nursing Association has derived substantial benefit from the drawing-room sale of work which was held last month on its behalf. Notwithstanding the unfavourable climatic conditions under which the sale took place, it realised £5 in excess of last year, and the Association has received a cheque for £48. In view of the growing popularity of the organisation, the desire to establish a nurses' home, which Mrs. Charles Ward, the founder and lady superintendent of the Association, has entertained from the outset, should be fulfilled at an early date. A second nurse is, however, the necessity of the moment.

### SUNDAY COLLECTIONS AT TUNSTALL.

The Committee of the Tunstall Town Nursing and Samaritan Society deplore the abandonment by many of the religious bodies of the practice which formerly obtained of having a collection once a year in aid of its funds, and at the annual meeting the hope was expressed that in future every place of worship would make it a point of fixing a convenient Sunday. We have no doubt that the request will be complied with if the members of the committee take a little trouble in the matter, make a few personal calls, and write a few personal letters. It must be recollected that the claims on the liberality of the churches and chapels are very numerous, and that those which are not pressed are very likely to be overlooked.

### NURSES AND MANAGEMENT.

At a meeting of the Stafford Board of Guardians a report presented by the Workhouse Visiting Committee in favour of placing the matron of the workhouse next in precedence to the medical officer, was discussed at some length. Mr. Peach avowed himself in sympathy with the recommendation of the Departmental Committee who suggested that the head nurse should have supreme control in the sick wards, and said that to give the matron power to go into the infirmary and to some extent to superintend the work of the head nurse, might lead to friction. Major Johnson, on the contrary, thought that the matron should be head so far as the management was concerned, and contended that although nurses did their own duties satisfactorily, they were no use as managers. This contention was promptly disposed of by Dr. Marson, who, speaking with the authority of practical experience, asked how the matron with no official training was to manage the infirmary better than the superintendent, who was officially trained. Nevertheless, the majority of Guardians adopted the view of Major Johnson. They will probably have to give way eventually, and Major Johnson will be afforded an opportunity of learning that the trained nurse can be at least as good a manager as the untrained matron.

### TINPLATE WORKERS AND NURSING.

At the annual meeting of the Neath Nursing Institute the hon. secretary was able to announce that the income for the year was £247 11s. and the expenditure £199 3s. Contributions had been received from many quarters, and one was a specially welcome subscription of three guineas from the men at the Eagle Tinplate Works. They also announced that they would be willing to quadruple the sum when the Association expands its operations. The Mayor of Neath, commenting upon this offer, said that he had endeavoured to the best of his ability to further the aims and objects of the Association, and he hoped that the people of Neath would increase their support and thus enable the institution to widen the scope of its good work. One nurse paid 3,072 visits to 123 patients, and the other 3,367 to 133 patients during the twelve months.

### CORNISH GUARDIANS AND DISTRICT NURSES.

Some time ago an endeavour was made by the Nursing Associations in the Redruth Union to induce the guardians to recognise the efficient practical work done by the nurses in the parishes included in the Union for the poor residents by a money grant. Although unsuccessful at the moment, the proposition has been evidently considered by the guardians, and when it was again mooted at their recent meeting, a resolution in favour of a contribution of two guineas per annum for each nurse engaged was carried, five of the Board only voting for an amendment that no subscription be given, on the ground that it would not be fair to parishes in which no nurses are employed. Eight nurses are at present engaged in this Union, which includes the chief mining centre of Cornwall.

### A NURSE AS TEACHER OF WOOD-CARVING.

It appears that Miss Wood, the nurse of the Bishop Auckland and District Workmen's Association, not only nurses the sick poor, but also acts as a teacher and a trainer. Some time ago, says a local paper, a young fellow met with a serious accident in the pit. He got his back broken, and will never be able to work again. To relieve the monotony of his life, the nurse set herself the task of teaching him crochet work and wood-carving. He has proved himself a very apt pupil, and several examples of both classes of his work have been very much admired.

### SHORT ITEMS.

The s.s. *Dunvegan Castle*, which arrived at Southampton on Saturday last, had on board nursing sisters E. A. Crouch, J. Mitchell, B. Rennie, and P. B. Watson, of the Army Nursing Service Reserve, who return from South Africa on the reduction of establishment.—A successful garden fête was held last week in the grounds of Mapesbury House, Kilburn, lent by Mr. Chester Foulsham, in aid of the Passmore Edwards Hospital at Willesden.

### The Nursing Outlook.

" From magnanimity, all fear above;
From nobler recompense, above applause,
Which owes to man's short outlook all its charm."

### FALSE TRAINING AND TRUE.

It is a fact that the falsely-trained nurse outnumbers the truly-trained nurse by three to one. This is taking as our standard of training three years—of which two must be spent in a hospital or infirmary of over 100 beds where lectures are given and examinations held.

There are various ways by which the so-called nurse adopts her role ; she can merely put on uniform and call herself Sister So-and-so, she can do six weeks' training in a maternity hospital, she can do three months' training at some such place as Plaistow, she can do three months in a big hospital as a paying probationer, or three months in an infirmary as a candidate for one of the religious societies that dress their district visitors as nurses and give them brief experience. Or there is a year's training to be had in certain cottage hospitals and nursing homes by paying for it.

No wonder the real nurse is crowded out of the field by the sham ! The worst wrong of all is probably done by the small hospitals which exact a premium, give a certificate, and probably give no training. The nurse in dress only is generally discovered soon enough by the doctor ; her operations are generally confined to selling quack medicines or more disreputable pursuits, and she is more or less an open fraud. But when a tiny cottage hospital aspires to train nurses and makes a profit out of the training by taking fees from the unfortunate women they deceive, a deliberate wrong is done to the patients and to the public alike. It is one of those things perpetrated in the name of charity that are a disgrace to the nation. It came out very strongly in the evidence given before the Lords' Committee on Metropolitan Charities, that some of the little hospitals were chiefly run to advertise some medical specialist, and it looks as though cottage hospitals may be occasionally run to advertise some chairman or pseudo-philanthropist. For anyone truly desirous of benefiting the human race would never leave the patients of a little hospital to the mercy of an untrained woman ; would never turn that woman out on the public with a certificate that is misleading ; would never take from that woman a fee for "training" when there was neither *materiel* nor experience available to train in any adequate sense.

Very much the same might be said of certain religious agencies that muddle up the salvation of the soul with the scientific care of the body. Because doors are open to the "nurse" that are not open to the "Bible-woman," a little experience in poultice-making and temperature taking is secured for the district visitor, and then a nursing dress is adopted, and prayers and poultices are run together. If a woman really cares enough for humanity to desire to save both souls and bodies, she should give three years to her nursing training, she should not descend to the deception of wearing a costume which tends to mislead those to whom she ministers. From the religious side of her mission truth in word and deed is a primary essential. And the fault is chiefly with those who thus train (?) her and send her forth ; what wonder that the woman, when her small salary and invidious position grow irksome, should enter a private nursing institution and pose henceforth as a fully-trained nurse ?

Again, in a keen desire to make money, and a careless disregard of that uncommon virtue usually called "common" honesty, some man or woman starts a nursing home, takes in probationers, they see only a few special cases, and yet after some months are often sent out as private nurses, and at the end of 12 months may be given a certificate. Here there is no question of charity at all, it is frankly a money-making and very often a sweating concern. Of course the nurse who allows herself to be used in this way is either a fool or a knave. Some of the small special hospitals, particularly maternity hospitals, train in a somewhat similar way—giving a certificate in a short time for the special branch of nursing they represent.

Finally, there is the training in district or cottage nursing, which is meant to be cheap so that the nurse can act as scrubber when necessary. This is a mistaken ideal, another delusion of so-called charity. The more out of the way a cottage, the poorer the patient, the more necessity for a fully-trained nurse who can recognise all symptoms and be equal to all emergencies. And the woman who has been a scrubber all her life is far less likely to turn to and do a thorough clean-out of a cottage, than is the hospital-trained nurse who appreciates scientifically the value of cleanliness, and who has been held responsible for the state of her ward by a stern and exacting matron. And there is no guarantee that your cottage nurse will keep to her village. She also, at times, tires of her low wage and wearisome work, and then she enters a nursing home and is sent forth to the public as a fully-trained nurse.

Here are some of the ways in which the nursing world has been flooded with flightily-dressed and badly-trained women. The presence of these women in the profession is a serious consideration. Later on we shall consider certain steps for reducing their number, and try to get the smaller hospitals and institutions to achieve a higher ideal. Meanwhile it may be pointed out, that there are ways in which these "special" fields of training might be made most valuable, instead of being misleading, if only we could get better organisation of the nursing profession. But at present it is not fair on our many very excellent training schools that do so much to supply thoroughly competent nurses, that these half-trained women should be supplied to the public which regards them as typical representatives of the whole profession.

### Lectures on Ophthalmic Nursing.

By A. S. COBBLEDICK, M.D., B.S.Lond., Senior Clinical Assistant and late House-Surgeon and Registrar to the Royal Eye Hospital.

### LECTURE XIV.—GRANULAR OPHTHALMIA: ITS SYMPTOMS, DIAGNOSIS, AND TREATMENT.

THE more common name for this affection is trachoma. It is, at first, more a disease of the inner surface of the eyelids than of the eye itself. As its name implies, a number of separate granular bodies varying in size, but usually about the size of a pin's head, make their appearance on the conjunctiva covering the inner surface, first, of the upper eyelid, and eventually of the lower lid also. If the trouble were confined to the lids it would not be a serious disease to deal with, but, unfortunately, the continual irritation caused by the granules moving over the smooth cornea sets up trouble in the latter, which may terminate by seriously impairing the vision, if not almost destroying it.

It is a disease which is chiefly confined to the poor classes, whose food is not too plentiful, where overcrowding is the chief feature of their home life, and where the surroundings are insanitary.

It occasionally breaks out in epidemic form in large institutions.

The disease is probably contagious, though some authorities deny this. Until there is conclusive proof on this point, I would advise all who have to deal with this condition—whether nurses or medical attendants—to take every precaution against infection. After examining a case, immerse the fingers in carbolic lotion 1 in 20, or some similar antiseptic, and follow this by washing in hot water.

The exact cause of this disease is unknown; it is more than likely that it is due to one or more bacillary bodies.

The symptoms at first are not very marked—a sense of grittiness or something in the eye. This is frequently accompanied by a slight drooping of the upper lid, giving the patient a tired appearance.

As the disease progresses, the patient complains of the eye "*watering*" freely and of inability to face a strong wind or glaring light. If the disease is not actively treated the cornea becomes next affected; a very characteristic condition termed *pannus* manifests itself: it consists of a dulling of the cornea in its upper third and the appearance of a large number of fine blood-vessels which can be seen traversing the cornea as far as the opacity extends. The corneal complication is, however, not always so characteristic in appearance, as separate transparent ulcers, which heal very slowly, may be caused by trachoma.

As the corneal opacity extends over the eye, the vision becomes much impaired, and adds to the patient's miserable condition.

*Prognosis.*—If the cases are diagnosed early and treated drastically, the cornea need not become involved. If an opacity of the cornea is once well established it is always certain to impair vision to some extent. If the inner surface of the lids has been too drastically treated, a scar may result, and as a consequence the edge of the lid is doubled in so as to bring the eyelashes on to the sensitive cornea: this condition is termed *trichiasis.*

*Diagnosis.*—Early cases are not always diagnosed unless the upper lid is everted as a routine practice. The granules if once seen are very typical, raised and somewhat gelatinous. The contents can be expressed by pressure. If the cornea is affected it does not always show typical *pannus*, so that it is wise to evert the upper lid in any case of corneal affection.

*Treatment.*—This consists in destroying the granules. As a rule caustics are used; the other alternative is to express the contents by means of a pair of roller forceps, (Fig. 1).

Of caustics the most common in use is silver nitrate. If the solid salt or the mitigated point is used, much care must be exercised, or the resulting scar will draw the eyelashes on to the cornea. If this treatment is followed, the patient should be under strict medical supervision.

FULL SIZE
FIG. 1.

The *mitigated caustic* consists of silver nitrate 1 part and potassium nitrate in 2, 3, or 4 parts: the two are fused together and moulded into small points.

Another preparation which in the past has had much vogue is *lapis divinus*, which consists of equal parts of copper sulphate, alum, and potassium nitrate.

Very good results are obtained by the daily application of a solution of the perchloride of mercury, 1 in 30 or 40 of glycerine and water, to the affected part. It should be well rubbed into the lid, after applying gutt. cocainæ 8 per cent. to the part, by damping a *firm* pledget of absorbent wool with the solution. As might be expected this is a painful process at first, but patients become tolerant.

Lead and copper salts are not very much used excepting in mild cases.

Expression with Knapp's roller forceps requires the administration of a general anæsthetic. In using this instrument the operator should constantly beware of allowing it to come in contact with the cornea.

Hot burning pain usully follows the operation, and is best relieved by a boracic flap frequently immersed in ice-cold boracic lotion.

This by no means exhausts the diseases to which the conjunctiva is prone, but the most common ones have been touched upon.

Time and space will not allow of more than mention of tuberculosis of the conjunctiva, of tumours, pemphigus, xerosis, and other morbid conditions. Common diseases of the cornea will be dealt with in the next lecture.

### To Nurses.

WE invite contributions from any of our readers, and shall be glad to pay for "Notes on News from the Nursing World," or for articles describing nursing experiences, or dealing with any nursing question from an original point of view. The minimum payment for contributions is 5s., but we welcome interesting contributions of a column, or a page, in length. It may be added that notices of appointments, entertainments, presentations, and deaths are not paid for, but that we are always glad to receive them. All rejected manuscripts are returned in due course, and all payments for manuscripts used are made as early as possible after the beginning of each quarter.

### The Application of a Linseed Poultice.

### EXAMINATION QUESTIONS FOR NURSES.

THE question was as follows:—If told to apply a linseed poultice, how should you proceed? Describe the process from arranging materials and implements to application to patient.

### FIRST PRIZE.

I should first ascertain that I had a sufficiency of boiling water, then put before the fire some linseed meal, a receiver, some cotton wool, and a towel; I should also fill a poultice bowl with boiling water, and into it put a spatula. I should then tease out some tow on a poultice board, according to the size of the poultice required, and then prepare my patient as much as possible and place in readiness a piece of jaconette and a broad flannel bandage, so that there might be no delay in applying the poultice when made. I should then empty the poultice-bowl and put into it the requisite quantity of boiling water to make the poultice of the required size, then quickly sprinkle in the linseed meal little by little, briskly stirring with the spatula in one direction all the time until the right consistence is attained, which should not be too firm but just sufficiently moist to turn out of the bowl without adhering to the sides. I should then rapidly spread the mixture on the tow to the thickness of half an inch, leaving a margin of about an inch and a half to be turned over the edges. I should dip the spatula in olive oil and pass it lightly over the poultice, just to cool the surface a little, not to prevent it sticking, for if the poultice is well made it will not stick to the patient or to itself when folded together. I should then carry it to the patient on the heated receiver, covered with the wool and towel.

Before applying the poultice I should carefully dry the skin with a soft warm towel; this is especially necessary if there is much perspiration to prevent irritation and the formation of papules. I should also test the heat of the poultice with the back of my hand before placing it on the skin. This precaution is particularly necessary if the patient is suffering from dropsy or paralysis owing to the extreme liability of such patients to become scalded on the application of heat, which, in other conditions of the body, could be quite comfortably endured.

After placing the poultice on the affected part I should well cover it with jaconette and warm wool, and secure the whole in position with a broad flannel bandage.

GLADYS.

### SECOND PRIZE.

To make and apply a linseed poultice, the first thing to do is to get all the materials together. Put the water on to boil, then get the linseed, bowl, spatula, and tow or flannel. When the water boils put a little into the bowl to make it hot, dipping the spatula in hot water also, pour this away and put in enough boiling water necessary for the poultice. Next, mix in the linseed, putting it in slowly with the left hand while stirring it with the right. When stiff enough, spread it on the tow (or flannel) about half an inch in thickness, leaving a margin all round to turn over the edges of the poultice.

Put the linseed next the skin for preference, but if the patient very much objects, put a piece of very thin muslin over.

When it is ready, roll up and wrap in a piece of hot flannel, or put between two hot plates to keep hot while being carried to the patient. Get the patient all ready to receive the poultice next. If it is a large poultice, a many-tailed bandage or a binder is the best to keep it in place (have all these ready at hand). Do not put on the poultice too suddenly, first try the heat on the back of the hand, and if you are able to bear it, you may safely put it on the patient. Keep the hands under the poultice and let it down gently, easing it a little if necessary before fastening it on. Never pop it on quickly, to cause shock. Cover the poultice with a piece of mackintosh to keep in the heat, then fasten on with the bandage (or binder), securing it with safety pins.

If the skin is very tender, put a little vaseline on the poultice. In renewing a poultice, always make the fresh one before taking off the old. In a case of jacket poultices, roll the patient on to his side, putting on the back first, roll him back again and put on the front one.

"KITTEN."

The reason of the prize-winner's success:—"Gladys" gained the first prize because she gives evidence of thoughtfulness and practical common sense in speaking of the dangers to be guarded against in cases of dropsy and paralysis; but in one respect her answer is not so good as that of "Kitten," the winner of the second prize, because she does not mention the very sensible mode of applying the poultice suggested by "Kitten," namely, that of keeping her hands at first between it and the patient's body, and removing them gradually. The latter nurse becomes a little mixed in her directions as to the order of procedure, but that comes from being unaccustomed to literary effort, not from non-comprehension of the subject.

Merits and defects of the papers generally:—An immense number of nurses have competed this month; everyone seems to have felt that they had something to say on the subject, and, broadly speaking, the answers are excellent. One slight error is that almost all have been unable to detach their minds from a jacket poultice, whereas the question made no such reference. However, if a jacket poultice can be successfully applied so can any other, for it is the most complicated form of the article. Only two competitors mentioned the occasional desirability of applying a poultice in a bag. It is not the correct way generally, but in cases of great restlessness it is helpful.

### HONOURABLE MENTION.

This is gained by "Mona," "Saque," Marie," and "Nurse D.," all of whom send excellent papers, especially "Nurse D."

No further questions till October. The session is now closed for the summer. The next question will appear the first week of October.

THE EXAMINER.

### Presentations.

CORBETT HOSPITAL, STOURBRIDGE.—Miss Kate E. Richards, on the occasion of leaving the Corbett Hospital, has been presented by the committee and medical staff with a handsome American roll top desk and an illuminated framed testimonial expressing their appreciation of her efficient work as matron. The nursing staff and household gave a brass inkstand and framed photographic group, etc.

HOPE HOSPITAL, PENDLETON, MANCHESTER.—Miss E. Musker was presented with a handsome timepiece from the nurses at Hope Hospital, Pendleton, on the occasion of leaving to take up her new post as nurse-matron at Birkdale Isolation Hospital.

### TRAVEL NOTES AND QUERIES.

CORRECTION OF TERMS IN PARIS (M. S.)—Thanks for valuable information. It is always pleasant to hear of the success of a trip.

VILLA NAPOLI (C. A. P.).—Thanks for change of address and valuable testimony to excellence of accommodation. It is so useful to have independent testimony on these matters.

THREE WEEKS IN FRANCE FOR £5 (Margaret).—You have misunderstood me—you cannot live in Candebec for three weeks and *get there* for £5. The cheapest hotel is De la Marine, which at 6 francs per day will be 5s. without tips—35s. per week. St. Servan is dearer, 7 francs per day. Knokke, in Belgium, would be cheaper and the journey only costs 12s. return.

### Queen Victoria's Jubilee Institute for Nurses.

Her Majesty Queen Alexandra has been graciously pleased to approve the appointment of the following as "Queen's Nurses," to date July 1st, 1903:—

ENGLAND.

| Nurse | District Training at | Working at |
|---|---|---|
| Agnes E. Edwards | Battersea | Shildon |
| Caroline Billinghurst | Bermondsey | Fletton |
| Mary A. Wilcox | Ditto | Newmarket |
| Mary E. Gibson | Birmingham | Birmingham |
| Florence E. Scott | Ditto | Ditto |
| Mary H. B. Empson | Bloomsbury | Kilburn |
| Kate Ferris | Ditto | London |
| Emily Lane | Ditto | Horsforth |
| Constance M. Moulder | Ditto | Hanley |
| Frances M. Rice | Ditto | Bloomsbury |
| Mary G. Searle | Ditto | Wormbridge |
| Ethel Tilbury | Ditto | New Malden |
| Alice M. A. Watson | Ditto | King's Lynn |
| Emily M. Whiteman | Ditto | Whittlesea |
| Eliza Davies | Bolton | Bolton |
| Jeannie Evans | Brighton | Gloucester |
| Ellen A. Hancox | Ditto | Brixton |
| Alice M. Knight | Ditto | Brighton |
| Sarah A. Mellor | Ditto | Garston |
| Alice R. Holden | Camberwell | Tunbridge Wells |
| Esther J. Millican | Ditto | Caton |
| Harriet Camp | Cardiff | Waterfoot |
| Jane Thompson | Ditto | Burnley |
| Alice M. S. Smith | Chelsea | Gosport |
| Grace Lilian Thomas | Ditto | Chelsea |
| Mary E. Parkhouse | Coventry | Coventry |
| Emma M. Coles | East London | East London |
| Edith M. A. Ramsay | Ditto | Ditto |
| Clara A. Hinde | Gloucester | Churcham |
| Mary E. John | Ditto | Carshalton |
| Eleanor M. Bounds | Hammersmith | Bury |
| Alice A. Fudge | Leeds (Holbeck) | Leeds |
| Beatrice M. Taylor | Ditto | Ditto |
| Hilda M. Taylor | Ditto | Ditto |
| Nellie West | Ditto | Ditto |
| Eva Pashley | Ditto (Lovell Street) | Ditto |
| Fanny R. Bacchus | Liverpool (Central) | Liverpool |
| Mary E. Powderley | Ditto | Ditto |
| Alice Prescott | Ditto | Ditto |
| Mary A. M. Fairhurst | Liverpool (Kirkdale Road) | Ditto |
| Florence J. White | Liverpool (Overton Street) | Ditto |
| Mary T. Cunningham | Liverpool (Shaw Street) | Ditto |
| Ethel M. Walby | Ditto | Ditto |
| Ada S. Bearpark | Manchester (Ardwick Green) | Manchester |
| Laura Fothergill | Ditto | Ditto |
| Eva E. Righton | Ditto | Ditto |
| Charlotte Dudley | Manchester (Bradford) | Ditto |
| Sarah A. Robinson | Ditto | Kensington |
| Minnie Burdon | Manchester (Harpurhey) | Manchester |
| Isabella Jackson | Ditto | Ditto |
| Hannah M. Newton | Ditto | Ditto |
| Annie Woods | Ditto | Ditto |
| Julia A. Hardy | Manchester (Hulme) | Ditto |
| May McMahon | Ditto | Ditto |
| Emma J. Watts | Ditto | Ditto |
| Sarah P. Watts | Northampton | Northampton |
| Kate Henrys | Notts. N. Federation | Balderton |
| Mary A. Jackson | Portsmouth | Portsmouth |
| Hylda M. Newman | Ditto | Hackney |
| Alice M. Moxhay | Reading | Reading |
| Winifred Froude | Salford | Salford |
| Catherine E. Waldock | Ditto | Bedworth |

ENGLAND—*Continued.*

| Nurse | District Training at | Working at |
|---|---|---|
| Marian Benbroke | Shoreditch | Gateshead-on-Tyne |
| Gertrude A. Gilby | Ditto | Gillingham |
| Susan W. J. Hadden | Ditto | Kenilworth |
| Jessie Matheson | Ditto | Rochdale |
| Louise Mitchell | Ditto | Bishop Auckland |
| Mary B. Pattison | Ditto | Shoreditch |
| Constance May | Southampton | Gloucester |
| Agnes Colvie Angus | Sunderland | Sunderland |
| Ellen Lyon Corser | Ditto | Ditto |
| Marion Amy Hall | Ditto | Ditto |
| Ellen Oliver | Walworth | Bilston |
| Harriet Sheridan | Westminster | Westminster |

WALES.

| Nurse | District Training at | Working at |
|---|---|---|
| Winifred Anne Jones | Bermondsey | Amlwch |
| Eleanor S. Campion | Brighton | Bangor |
| Sarah M. Haswell | Cardiff | Cardiff |
| Jane Anne Laurence | Gloucester | Conway |
| Margaret A. Bushell | Portsmouth | Ystalyfera |
| Alice M. Hawes | Ditto | Cardigan |
| Katherine Howell | Salford | Swansea |
| Mary A. Jones | Shoreditch | Pontypridd |
| Gertrude E. Clowes | Treorchy | Treorchy |
| Catherine M. Parry | Walworth | Bethesda |

SCOTLAND.

| Nurse | District Training at | Working at |
|---|---|---|
| Jessie A. Baxter | Edinburgh | Innerleithen |
| Margaret Blyth | Ditto | Edinburgh |
| Annie M. Brown | Ditto | Gourock |
| Isabella Bryden | Ditto | Greenock |
| Jean Cameron | Ditto | Alexandria |
| Annie Chalkley | Ditto | Kinneil |
| Jessie C. Forsyth | Ditto | Edinburgh |
| Charlotte McCallum | Ditto | Argyllshire |
| Mary Millar | Ditto | Cowdenbeath |
| Lydia B. Nesbitt | Ditto | Edinburgh |
| Isabella Robertson | Ditto | Edinburgh |
| Annie M. Scott | Ditto | Lochwinnoch |
| Sydney Sinclair | Ditto | Alexandria |
| Margaret F. T. Skene | Ditto | Edinburgh |
| Annie Smibert | Ditto | Edinburgh |
| Helena L. Smith | Ditto | Perth |
| Douglas Stevenson | Ditto | Lochwinnoch |
| Mary Stewart | Ditto | Dalmeny |
| Helen Thorburn | Ditto | Shielbridge |
| Helen B. Wilkie | Ditto | Edinburgh |
| Annabella Campbell | Aberdeen | Aberdeen |
| Helen E. Smith | Ditto | Ditto |
| Jane B. McAllister | Glasgow | Glasgow |
| Isabel C. Miller | Ditto | Ditto |
| Margaret Peterkin | Ditto | Ditto |
| Nancy F. Wilson | Ditto | Ditto |

IRELAND.

| Nurse | District Training at | Working at |
|---|---|---|
| Marie Finn | St. Lawrence's, Dublin | Dublin |
| Louise Gillespie | Ditto | St. Marnock's |
| Elizabeth H. McCoy | Ditto | Dublin |
| Elizabeth G. Hore | Ditto | Rathfarnham |
| Louisa Trinham | Ditto | Ballycroy |
| Aileen L. Keogh | St. Patrick's Dublin | Powerscourt |
| Annie Richardson | Ditto | Dublin |
| Elizabeth W. Tate | Ditto | Ditto |

### Resignations.

Miss S. R. Alice Rimmington has resigned her position as lady superintendent at Bancroft's School, Woodbridge Green, Essex, and has taken a training home at Brighton in Regency Square, which she will open on August 1st. Miss Rimmington was formerly matron of the Nottingham General Hospital, and is well known in the nursing world.

### Everybody's Opinion.

[Correspondence on all subjects is invited, but we cannot in any way be responsible for the opinions expressed by our correspondents. No communication can be entertained if the name and address of the correspondent are not given as a guarantee of good faith, but not necessarily for publication. All correspondents should write on one side of the paper only.]

### THE ONE-YEAR NURSE.

Mrs. Richmond, Matron of Luton Workhouse, writes: Will you allow me to say that nothing in Miss Wilson's letter or that transpired at their conference has in the least shaken my faith in the ultimate good and not widespread harm which will result from the organisation of minor schools? Also, that I am astounded that anyone putting forward such a plan as the one characterised as a "national one" should call it practical. Another point dealt with at their conference was whether or not it was desirable that the matron of the workhouse should be a trained nurse and hold the joint appointment of matron and superintendent nurse. Now, in dealing with such an important point as the qualification of a matron, is it too much to ask for the treatment to be just? And one inspector being quoted as having said that he considered the two offices incompatible, it would only have been fair to have added that if opposed by one inspector it was supported by several, and very strongly by one who said that in his district out of 47 matrons, 26 were trained nurses; that for years he had suggested to guardians, when appointing matrons, to give the preference to trained nurses, and that he had every reason to be satisfied with his advice. Surely in the face of these facts, and the conspicuous benefit and "not serious consequences" which the evidence proves to be the result of the practice, the Workhouse Nursing Association cannot hope to induce the president of the Local Government Board to set aside this recommendation of his committee.

### LONDON AND MELBOURNE NURSING HOMES COMPARED.

Miss E. Glover, one of the Joint Hon. Secretaries of the Victorian Trained Nurses' Association, writes to us under date of March 9th, in reference to an article on "Pay Hospitals" in The Hospital of January 10th as follows:—Complaints are frequently made by Australian visitors to London of the exorbitant prices charged, and indifferent accommodation provided in the private nursing homes as compared with those we have in this country. My hospital cost me £6,000 (borrowed) to purchase, £1,000 in alterations under the watchful eye of the Board of Health, fitting up operation room, etc., £1,000 more to furnish. My fees are from £3 3s. to £6 6s. per week, very rarely £6 6s. and I am pleased to say the first year shows very creditable profit. There is only accommodation for 18 patients; dressings, etc., are charged slightly over cost price, and only in exceptional cases, or where the patient can afford the luxury, is a special nurse put on. My nurses' (all certificated) salaries and servants' wages cost nearly £700 per annum. I cannot see why there should be any necessity to appeal to the public to support hospitals for patients who can afford to pay a moderate fee. A loan at the usual rate of interest would surely suffice for a nurse with any ordinary capacity for hospital administration, especially if she be supported by the best men in the profession. I do not suppose middle-class persons would like to accept as charity what they are really paying more than enough for. I am afraid the nursing world has not been giving sufficient time to the study of hospital management; economy seems the most neglected of all the virtues. It is only by comparison that we can form any judgment of the administrations of these private institutions. I have a great scheme in view now to build a large residential club for private nurses. I do not want any gifts towards it, but I am hoping to find some wealthy person who will lend us a sufficient sum upon which we will pay interest, taking the building and furniture as security. The money can gradually be paid off, and we shall stand proudly on our own efforts. Now that we are insisting upon a trained nurse being put at the head of all nursing institutions, I think we must also insist upon all nurses being taught, what you rightly term in one of your papers, the "Higher Branches of Nursing."

### THE SOURCES OF SUPPLY.

A Correspondent writes:—The article on "The Sources of Supply" from which nurses are drawn leaves an impression not intended most probably by the writer, viz., that gentlewomen who enter the nursing profession are either "sentimental damsels" or "the most unsatisfactory of their sex the poverty-stricken ladies" narrowed and useless. There are, however, plenty of women of gentle birth and breeding in hospitals, districts, and doing private nursing, who are possessed of capability, organising power and skill, in addition to a true love and devotion to their patients and their work. Why decry these women and belaud the "housemaid class" at their expense? Both do good work and there is plenty of room for both in the nursing profession. But everyone will agree that the nursing profession would be better without the "genteel young lady" who is a trial to everyone during her training and who blossoms later into the pretentious snob who is always explaining that there is "no need" for her to be a nurse, and who tries to demonstrate to everyone that she knows nothing of dusting or sweeping, because she is "a lady;" a fact one certainly would never have discovered for oneself. But social considerations cannot be ignored in a hospital any more than in any other place. Certainly hospital training should be open to all who possess good health and character, aptitude for the work, and a certain standard of education. But there I would stop. Hospital appointments should be given to gentlewomen. The head of a ward must possess, in addition to the essential qualifications of a good nurse, *i.e.*, sympathy, conscientiousness, and common sense, much *savoir faire*, a cultivated mind, and unfailing tact and courtesy, besides the organising and teaching powers required of a ward sister. She must be able to maintain a proper tone in her ward and preserve the *juste milieu* between familiarity on the one hand and frigidity on the other, in the relations of staff, students, nurses and patients in her little world. In a word she must be a well-bred woman, and her staff nurse (an embryo sister) must be one too. Where the heads of a ward are of much the same class as their patients the tone at once becomes "free and easy," especially in a male ward. This was very noticeable during the war, where all sorts and conditions of trained and untrained or partly-trained women acted as ward sisters. It is also well known to night sisters who have the opportunity of observing how the tone of the same ward will alter with its night nurse and who can compare various wards and see the influence of a well-bred woman reflected in the manners and customs of the nurses and patients in the ward under her care.

### Where to Go.

Monday, July 13.—At 3 p.m., performance by the Old Comedy Society, of three pastoral plays, in the grounds of Lowther Lodge, Kensington Gore, in aid of the London Hospital.

Thursday, July 30.—Festival Service in Godalming Parish Church at 3 p.m., and garden party in the grounds of the Meath House of Comfort, at 4 p.m. (anniversary meeting).

### "The Hospital" Convalescent Fund.

The hon. sec. begs to acknowledge with many thanks the receipt of 2s. 6d. from "E. C. N."

### Wants and Workers.

The Hospital.—Nurse M. A. Godwin would be glad if any reader of The Hospital, who takes it regularly and has no further use for it, would kindly send it on to her at 25 Vincent Road, Dorking, on either the Monday or Tuesday after publication. Stamped addressed wrappers would be forwarded, quarterly in advance.

### Echoes from the Outside World.

### Birth of an English Prince.

LAST Friday it was officially announced that on the previous evening, at 5.50, her Royal Highness Princess Charles of Denmark had given birth to a son. Princess Charles of Denmark is better known as Princess Maud, and is the third daughter and youngest child of the King and Queen. She was born in 1869, and was married in 1896 to Prince Charles, who is her cousin. Prince Charles is fifth in the line of succession to the throne of Denmark. He and his wife reside at Appleton House, which has a delightful outlook over Sandringham Heath to the pine woods. In order to be within easier reach of her daughter, the Queen was temporarily accommodated at the agent's house at Sandringham. Princess Maud and the young Prince are both making favourable progress.

### The Visit of the President of the French Republic.

ON Monday, M. Loubet, President of the French Republic, arrived in England, and was received at Dover by the Duke of Connaught and the French Ambassador, the British Fleet firing a salute as he stepped ashore. After he had been presented with an address by the Mayor, he was conducted to the Royal carriage and driven to the station, the route being lined by troops. At Victoria Station our visitor was received by a brilliant and representative gathering, including the King, the Prince of Wales and other members of the Royal family, several Cabinet Ministers, and the Commander-in-Chief. On alighting he was cordially greeted by his Majesty, and after a brief interval entered an open carriage in company with the King, the Prince of Wales, and the Duke of Connaught, the distinguished party being loudly cheered by crowds of people on their way to St. James's Palace. Subsequently, the President made formal calls on the King and Queen, the Prince and Princess of Wales, and the Duke and Duchess of Connaught, and, having received a deputation of the French community in London at the French Embassy, he and his suite dined with their Majesties at Buckingham Palace. The King, in proposing the President's health, said that France, being nearest to England, ought to be her best neighbour; and M. Loubet, in reply, referred to the King's visit to Paris, which, he was sure, would have the happiest effects, and would serve to maintain and draw still closer the relations between the two countries. On Tuesday, after visiting the French Hospital and the French Governesses' Home, and receiving deputations at the French Embassy, M. Loubet was the guest of the City at the Guildhall. The President had a magnificent reception from a distinguished company, which included several members of the Royal family. In responding to the address of welcome presented to him in the library, M. Loubet said that he joined heartily in the wishes expressed for the cordial understanding between two peoples, each of which held a necessary place in the history of civilisation. He was afterwards entertained at luncheon. In the evening the President entertained the King and Prince of Wales to dinner at the French Embassy, and the day closed with a State performance in his honour at the Royal Opera. On Wednesday M. Loubet visited Windsor, attended the review at Aldershot, dined with Lord Lansdowne, and was present at the State Ball at Buckingham Palace. His visit terminated on Thursday.

### M. Loubet's Career.

THE President of the French Republic, M. Emile Loubet, is the son of a small farmer, who was born on December 31st, 1838, and in accordance with his father's wishes became an advocate. He spent his student days in the Latin Quarter, but when he had taken his degree he began to practise at Montélimar, a few miles from his birthplace. His career has been marked by steady progress. In 1870 he was elected Mayor of Montélimar, and in 1876 Deputy. In 1885 he became a Senator, and in 1887 he was appointed Minister of Public Works. Five years later he became Prime Minister and Minister of the Interior, and in 1895 President of the Senate. In February, 1899, on the sudden death of M. Faure, he was elected President. Personally, M. Loubet has a very natural manner, he is a great reader, a hard worker, a confirmed smoker, and fond of music and art, whilst his ambition is said to be to settle down eventually on the old farm at Marsanne, where his mother still lives. His wife has the same simple tastes as himself, but shares with him to the fullest extent the honours of the existing rule at the Elysée. He has two sons and one daughter.

### Royalty at the Children's Fête.

ON Tuesday the Queen and Princess of Wales, the former accompanied by Princess Victoria and Princess May of Wales, and the latter by Princes Edward and Albert of Wales, visited the children's fête in the gardens of the Royal Botanic Society in aid of the funds of the National Society for the Prevention of Cruelty to Children. The Queen's visit was not the less appreciated because it was unexpected, and her Majesty's appearance excited great enthusiasm. The Royal party were received by Lord and Lady Ancaster and escorted by a number of young ladies with floral wands to the dell at the west side of the large conservatory where a raised pavilion had been specially erected. A number of dances, arranged by various ladies, took place, including a rose ballet, under the supervision of Lady Newton, and the entertainment was a brilliant success.

### Serious Illness of the Pope.

ON Friday last the Pope, while driving out, complained of a sudden feeling of *malaise*, and returned to his apartments, retiring immediately to bed. His physician remained all night at the Vatican in attendance. On Saturday an effort was made to allay the apprehensions of the public by means of an official bulletin, which stated that owing to the fatigue of his recent receptions his Holiness had consented to take a complete rest for a few days, but was otherwise in his usual health. This reassuring intimation was, however, followed on Sunday by a bulletin to the effect that the Pope was suffering from senile pulmonary hepatisation, and that, though his general condition in view of his age was serious, there was no immediate cause for alarm. Subsequent bulletins indicated that the weakness had slightly increased. But on Tuesday an operation was performed. The Pope bore it well, and according to the bulletin issued on the evening of that day the functions of circulation and respiration were slowly but gradually recovering themselves.

### Meeting of the Victoria League.

THE members of the Victoria League, an institution founded in memory of the late Queen, and having for one of its objects the promotion of closer union between the Mother Country and the Colonies, met on Thursday last week at the official residence of the First Lord of the Treasury, in Downing Street, the Countess of Jersey in the chair. There was an interesting discussion on Imperial Education, and among the speakers was Miss Balfour, sister of the Prime Minister. Miss Balfour illustrated the need for a further knowledge of the different parts of the Empire among those who belong to it, by the fact that at the beginning of the Boer war a lady in Manchester, supposed to be well educated, asked her if the Boers were Mohammedans.

### Appointments.

ALEXANDRA HOSPITAL, QUEEN SQUARE, LONDON.—Miss Isabella Murdoch has been appointed staff nurse. She was trained at Oldham Infirmary, and has since been staff nurse at the Royal Hospital for Sick Children, Edinburgh. She was a member of the Army Nursing Service Reserve from March 1900 to May 1903.

ATCHAM WORKHOUSE INFIRMARY. — Miss Katherine Morgan has been appointed charge nurse. She was trained at Crumpsall Workhouse Infirmary, and has since been nurse at Penrith Union Infirmary, assistant nurse at Lichfield Union Infirmary, and superintendent nurse at Haslingden Union Infirmary. She has also done private nursing in Manchester.

BIRKDALE ISOLATION HOSPITAL.—Miss E. Musker has been appointed nurse-matron. She was trained at Salford Union Infirmary, and has since been assistant nurse at Grafton Street Fever Hospital, Liverpool, charge nurse at Middlesbrough Union Infirmary, charge nurse at Stockton Fever Hospital, staff nurse at Leeds Hospital for Women and Children, and sister at Hope Hospital, Pendleton, Manchester.

BIRMINGHAM CITY HOSPITAL.—Miss Ellen J. Griffiths has been appointed ward sister. She was trained at Bradford Union Infirmary and Worcester Fever Hospital. She has since been charge nurse at Sunderland Union Infirmary.

FIR VALE INFIRMARY, SHEFFIELD.—Miss Millicent Tait has been appointed home sister and assistant superintendent. She was trained at the Royal Infirmary, Sheffield, and was afterwards staff nurse, sister, and deputy night sister. She has also done private nursing in London and Sheffield.

GORDON HOSPITAL FOR FISTULA, LONDON.—Miss K. Maud Moore has been appointed matron. She was trained at St. Bartholomew's Hospital, London, and has since been sister at the Hospital for Sick Children, Great Ormond Street, London, and matron of the Convalescent Branch; matron of the Royal Hospital for Women and Children, London; lecturer for the London School Board; and district nurse at Whitechapel.

KILTON HILL INFIRMARY, WORKSOP.—Miss Edith M. Barrand has been appointed matron. She was trained at Fir Vale Infirmary, Sheffield, where she has since been charge nurse. She has also been sister at the City Fever Hospital, Sheffield, and superintendent nurse at Bramley Union Infirmary, Armley, Leeds. She holds the L.O.S. certificate.

LEWISHAM INFIRMARY.—Miss Emily Duncan has been appointed ward sister. She was trained at St. George's Hospital, London.

MARGATE COTTAGE HOSPITAL.—Miss Florence Winch has been appointed staff nurse. She was trained at St. Saviour's Infirmary, East Dulwich, having previously been nurse at the Darenth Schools for Imbecile Children.

PERSHORE ISOLATION HOSPITAL.—Miss Mary Jane Hopton has been appointed nursing superintendent. She was trained at St. Marylebone Hospital, London, and has since been nurse at the Central Sick Asylum, Hendon; the Poplar and Stepney Sick Asylum, and nurse-in-charge at Pershore Temporary Isolation Hospital.

ROYAL VICTORIA HOMES, BRENTRY, NEAR BRISTOL.—Miss Agnes Cottle has been appointed matron. She was trained by the Cheltenham Nursing Association at the Royal Hants County Hospital, Winchester, and has since been sister at Fulham Infirmary, Hammersmith, and assistant matron at the Royal Victoria Homes, Brentry.

STEYNING WORKHOUSE INFIRMARY.—Miss Grace Wood has been appointed assistant superintendent and charge nurse. She was trained at Camberwell Infirmary, and has since been assistant nurse at Yarmouth Union Infirmary.

### Notes and Queries.

**REGULATIONS.**

**The Editor is always willing to answer in this column, without any fee, all reasonable questions, as soon as possible.**

**But the following rules must be carefully observed:—**

1. **Every communication must be accompanied by the name and address of the writer.**
2. **The question must always bear upon nursing, directly or indirectly.**

**If an answer is required by letter a fee of half-a-crown must be enclosed with the note containing the inquiry.**

*Honorary Staff.*

(132) Will you please tell me the names of the honorary members of the medical staff of the West End Hospital for Diseases of the Nervous System, 73 Welbeck Street, W.?—*M. E. C.*

You could ascertain by writing to the Secretary of the hospital, or by consulting "Burdett's Hospitals and Charities."

*Naval Nursing Service.*

(133) Will you kindly tell me to whom to apply for particulars respecting Queen Alexandra's Naval Nursing Service?—*Nurse.*

Apply to the Director-General, Medical Department of the Navy, Admiralty, Northumberland Avenue, W.C.

*L.O.S.*

(134) Will you kindly tell me what are the regulations of the L.O.S.? Having had practice of midwifery under a doctor, I do not wish to waste money and time in taking a regular course of instruction.—*A. T.*

Write and ask the Secretary, the London Obstetrica Society, 20 Hanover Square, London, W.

*Queen Victoria's Nursing Funds.*

(135) I should like to know for what purpose the Queen Victoria Jubilee and Memorial Funds are used. Is it for training Queen's nurses, or for payment of their salaries in the districts where they work, or for pensions?—*Bart's.*

The primary object of the Queen Victoria's Jubilee Institute for Nurses is to train women for the purpose of nursing the sick poor in their own homes.

*Home.*

(136) Can you tell me whether there is any home or institution where an incurable adult would be received on the payment of about 12s. weekly?—*F. N.*

You do not say whether you want the home for a man or a woman. See "Burdett's Hospitals and Charities" for a list of "Homes for Incurables."

*Hot Sweat.*

(137) I am about to go as a hospital nurse, but I am sorry to say that sometimes, for two or three days together, a hot sweat comes in the palms of my hands, and I have, at times, been hardly able to push my needle through my work. Will you kindly tell me if there is any cure?—*Alma.*

You must consult a medical man.

*Up-Country Nursing Association.*

(138) Will you kindly give me the particulars of the Up-Country Nursing Association?—*Simla and D. L.*

Write to Mrs. Sheppard, 10 Chester Place, Regent's Park, N.W.

*Medical Nursing Homes.*

(139) Will you kindly tell me of some medical nursing homes in Bournemouth? I cannot find what I want in "The Nursing Profession: How and Where to Train."—*Nurse F.*

We do not recommend private institutions. Search the local papers if you do not know anyone who can inquire locally for you.

*Patients.*

(140) Can you tell me how to get patients in my own home, which is comfortable and near London? I have been advertising in THE HOSPITAL on and off for some time, but have had no answers.—*Anxious.*

Try advertising in your local papers, and call upon, or send your recommendations to, the local medical men.

*Suppositories.*

(141) Will you kindly tell me if suppositories are as safe and effectual a remedy for constipation as enemas? If so, are there any particular sorts for the purpose?—*Welsh.*

Refer to the medical man in charge of the case.

*Male Nurses.*

(142) 1. What training do male nurses undergo? 2. Would holding a St. John's Ambulance certificate enable me to obtain employment? 3. What remuneration do male nurses get?—*Ammon-Chlor.*

1. Write to the National Hospital for the Paralysed and

Epileptic, Queen's Square, Bloomsbury, W.C. 2. Not sufficient. 3. The fees vary from £2 2s. to £4 4s.

*Pretoria.*

(143) I should be very glad if you could kindly tell me if there is any chance of a trained nurse obtaining work at Pretoria.—*Daisy.*

Write to the Hon. Secretary, the South African Expansion Emigration Committee, 47 Victoria Street, S.W., or the Matron of the Government Hospital, Pretoria, might kindly answer you. Enclose stamp. You should have maternity training.

*Maternity Law.*

(144) I should be glad to know if persons, not certificated, can continue to follow their work as monthly nurses as they have done for so long. I think that the new Act does not come into force until 1905. Please answer this.—*W. M.*

The new Act applies to midwives, not to monthly nurses. See article on the Midwives Act, 1902, in *Nursing Section,* May 9.

*Thyroid Gland.*

(145) Is there any danger in taking each day a 5 or 10 grs. tabloid of thyroid gland? I know some one who does it in order to decrease her weight, and she tells me that doctors order this remedy for that purpose.—*X. P. W.*

It is always risky to take drugs unless ordered by a doctor.

*Hospital Training.*

(146) Could you tell me of a hospital near Liverpool where I could get a year's training in surgical work? I find that most hospitals do not care to give one year's training.—*R. O'B.*

We cannot undertake to say that any hospital would take you for surgical work only, even as a paying probationer. You must write your requirements to the matrons of the hospitals, or you can advertise.

Can you tell me of a London or good provincial hospital where a year's general training can be obtained free or for a small fee?—*Aniline.*

Your only chance of obtaining general training for one year without fees is in one of the smaller hospitals which are not generally recognised as training schools. For such an appointment, advertise.

*Registration.*

(147) Would you tell me if there is a system of registration of trained nurses in England similar to that in New South Wales?—*E. M. B.*

No.

*District Training.*

(148) My father is the pastor of a large country parish, and the local doctor has offered to give me a year's training in nursing and a certificate at the end of that time. This training would enable me to help my father, but I should like to know if it would be sufficient to qualify me to hold responsible nursing posts.—*D. M.*

No, you would not be eligible for any position as a trained nurse. If you can be spared from home and like nursing, go to a good general hospital and train for a three years' certificate. See "The Nursing Profession: How and Where to Train" for full particulars.

*Incontinence.*

(149) Will you kindly tell me if there is any home which would take a little girl suffering from incontinence? She has been discharged from a London hospital as incurable. Her mother is a very poor widow, who would like her child to be sent where she would receive elementary education.—*Weybridge.*

Write to the Secretary, the "Invalid Children's Aid Association," 18 Buckingham Street, Strand, W.C.

*Special Training.*

(150) Will you kindly tell me where I can get a short training in nursing diseases of the heart? I am a maternity nurse, and have been asked to nurse so many heart cases that I should like to know more of the subject.—*S. B.*

Possibly you might come to some arrangement with the Matron of the City of London Hospital for Diseases of the Chest, Victoria Park, London, E., where patients suffering from heart disease are also received. There is also the National Hospital for Diseases of the Heart, Soho Square, W.C.

**Important Nursing Textbooks.**

"The Nursing Profession: How and where to Train." 2s. net; 2s. 4d. post free.

"A Handbook for Nurses." (New Edition). 5s. net; 5s. 4d. post free.

"The Human Body." 5s. post free.

"Ophthalmic Nursing." (New Edition). 3s. 6d. net; 3s. 10d. post free.

"Gynæcological Nursing." 1s. post free.

"Art of Feeding the Invalid." (Popular Edition). 1s. 6d. post free.

"Practical Hints on District Nursing." 1s. post free.

### For Reading to the Sick.

### OUR DARK DAYS.

I SAID, "the darkness shall content my soul;"
God said, "Let there be light."
I said, "the night shall see me reach my goal;"
Instead came dawning bright.

I bared my head to meet the smiter's stroke;
There came sweet dropping oil.
I waited trembling, but the voice that spoke
Said gently, "Cease thy toil."

I looked for evil, stern of face and pale;
Came good, too fair to tell:
I leaned on God when other joys did fail,
He gave me these as well.

*S. Williams.*

As long as we live, we shall be liable to alternating periods of joy and gloom. They may be long or short, severe or light; but they are sure to come, and they come to all. "The days come of which we say: They please me not." They are a searching trial, and a keen test of our love for our Lord; yet on those days He seeks fruit at our hands as much as on the sunny days, and we must not "send Him empty away." Often these dark days come without warning, perchance quite contrary to expectation, and we cannot account for them at all. If we are well, and able to distract ourselves, it is more easy to grapple with the clouds; but when we are ill, in bed all day, in pain all day, unable to read or work and disinclined for prayer, to face and profit by them is far from easy, yet noble and possible.

Learn from this how our Lord is generous beyond hope or thought to those who suffer with Him. He leaves them on their cross, perchance, but He is at their side, and His reward comes a hundredfold. Next, let the sick and desolate adopt and make their own the prayer of the good thief. When in pain, when a darkness of soul as that of Calvary seems to enshroud you and hide all light and joy from your eyes, cry out to our Lord who is very near, "Lord, remember me!" There is such pathos and simple humility in that word "remember." "Remember me!" And the answer will swiftly come and sink deep into your heart: "One day thou shalt be with Me in Paradise," for "if we suffer with Christ, we shall also reign with Him."—*Anon.*

Let him who gropes painfully in darkness or uncertain light lay this precept well to heart: "Do the Duty which lies nearest to thee" which thou knowest to be a Duty. The Second Duty will already have become clearer.—*Carlyle.*

Only strive to say, "My Saviour,"
As thou liest at His feet;
He can from thy dust and ashes
Spotless holiness complete.

Through the new strange stillness round thee,
Through the palpitating air,
A new dawn will steal upon thee;
How, thou canst not tell, nor where.

Piercèd hands will touch and bless thee,
Words descend from highest heaven,
Breathing through thy heart's recesses,
"O My child, thou art forgiven!"

*C. M. Noel.*

## Figures and Tables

**Fig. 1. f1:**